# A new species of *Oomyzus* Rondani (Hymenoptera, Eulophidae) reared from the pupae of *Coccinella septempunctata* (Coleoptera, Coccinellidae) in China

**DOI:** 10.3897/zookeys.953.53175

**Published:** 2020-07-27

**Authors:** Hai-Tian Song, Ming-Hui Fei, Bao-Ping Li, Chao-Dong Zhu, Huan-Xi Cao

**Affiliations:** 1 Department of Entomology, College of Plant Protection, Nanjing Agriculture University, No. 1 Weigang Rd, Nanjing, Jiangsu 210095, China; 2 Fujian Academy of Forestry, Fuzhou, Fujian 350012, China; 3 Key Laboratory of Zoological Systematics and Evolution, Institute of Zoology, Chinese Academy of Sciences, 1 Beichen West Road, Chaoyang District, Beijing, 100101, China; 4 National Animal Collection Resource Center, Institute of Zoology, Chinese Academy of Sciences, 1 Beichen West Road, Chaoyang District, Beijing, 100101, China

**Keywords:** Chalcidoidea, lady beetle, new taxon, parasitoid wasps, taxonomy, Tetrastichinae

## Abstract

*Oomyzus spiraculus* Song, Fei & Cao **sp. nov.** (Hymenoptera, Eulophidae) is described and illustrated as a gregarious larval-pupal endoparasitoid of *Coccinella septempunctata* L. (Coleoptera, Coccinellidae). Differentiation between *O. spiraculus* and its similar species is discussed and a key to differentiate the female and male of these species is provided. DNA barcodes of *O. spiraculus* and *O. scaposus* are analyzed and compared.

## Introduction

The seven-spotted lady beetle, *Coccinella septempunctata* L. (Coleoptera, Coccinellidae), is widely recorded from the Palearctic and has a large distribution in China. It plays a significant role as an effective predator by suppressing populations of homopteran pests (e.g. aphids, whiteflies, and scales), which cause severe damage to agricultural crops (Pervez 2002). It is attacked by multiple parasitoids from Hymenoptera and Diptera (Honet et al. 2019).

Because of the ecological and economic significance of *C. septempunctata*, the interactions between *C. septempunctata* and its parasitoids have been traced and studied for many years (Li 1984; Semyanov 1986; Schaefer and Semyanov 1992; Triltsch 1996; Ceryngier et al. 2012). Among these parasitoids, *Oomyzus scaposus* (Thomson) is a common parasitoid wasp of coccinellids (Song et al. 2017). During a recent project related to interactions between coccinellids and their parasitoids, *O. scaposus* and another *Oomyzus* species were reared from the pupae of *C. septempunctata*. Here, this other *Oomyzus* species is described as new to science and compared to other known *Oomyzus* species.

*Oomyzus* is one of the smaller genera in Tetrastichinae (Hymenoptera, Eulophidae), with 26 described species prior to this study. Graham (1991) revised the European species of *Oomyzus* and included keys to the females of 12 species and males of 11 species. Although some species of *Oomyzus* (e.g. *O. gallerucae* (Fonscolombe) and *O. sokolowskii* (Kurdjumov)) have been recorded as cosmopolitan and some others have a large distribution across different continents owing to biocontrol introductions (e.g. *O. brevistigma* (Gahan)) (Noyes 2019), most species are rarely found or recorded from more than one zoogeographical region. This is possible because *Oomyzus* is difficult to characterize and shares some features with some other genera of Tetrastichinae, such as *Baryscapus* Förster, *Tetrastichus* Haliday, and *Quadrastichus* Girault. Species of *Oomyzus* are mainly larval or pupal parasitoids of Coleoptera, sometimes of Lepidoptera, Neuroptera, and Diptera, and sometimes as egg parasitoids of their hosts (Graham 1991). Some species, such as *O. incertus* (Ratzeburg), *O. brevistigma*, and *O. sokolowskii*, have been successfully used as biological control agents against some important agricultural pests of leaf beetles (LaSalle 1994).

Graham (1991) preliminarily discussed assignments of species groups for some European species of *Oomyzus* based on morphological studies. However, to confirm these species groups further evidence is required. Although DNA barcode fragments of *O. scaposus* and *O. spiraculus* were generated in this study, the assignment to species group is not included because of the absence of data for the other species. Therefore, only COI fragments of these two species were analyzed and compared, even though they do not seem very close using morphological data. Despite the lack of molecular data, the differences and similarities of this new species between some possibly close species are discussed based on morphology.

## Materials and methods

### Parasitoid wasp collection and rearing

The pupae of *C. septempunctata* were collected during field surveys in Nanjing, China, 2018. The host pupae were placed in plastic cups covered with mesh cloth and moved to the Laboratory of Biological Control in Nanjing Agricultural University and maintained in an insectary (25 ± 1 °C, 60 ± 5% RH, photoperiod L16: D 8 h) to rear adults of lady beetles or parasitoid wasps. Emerged wasps were then used to establish colonies using healthy larvae of *C. septempunctata*. Sample individuals from reared colonies were preserved in 95% ethanol after emergence for further use.

### Taxonomy

Specimens used for morphological studies were critical-point dried with a Leica EM CPD300 automated critical point dryer. Then some specimens were mounted on cards and some others were dissected into head, mesosoma, metasoma, and gaster for scanning electron microscopy (SEM). Specimens were examined using a Nikon SMZ 1500 stereomicroscope fitted with a 10 mm ocular grid having 100 divisions. Habitus pictures were taken with a Nikon D7000 digital camera connected to the stereomicroscope. Dissected parts used for SEM were sputter-coated with gold using a Leica EM SCD050 super cool sputter coater. Micrographs were taken using an FEI Quanta 450 environmental scanning electron microscope. Photographs of appendages (fore wings, antennae, and legs) were taken with a Canon 550D digital camera connected to a Leica DM-2500 compound microscope. All color pictures were stacked using Helicon Focus software. The images were processed and combined into plates using Adobe Photoshop CC 2015.

The terminology follows Gibson (1997). Abbreviations are as follows: F1–F3, funiculars 1–3; MLM, midlobe of mesoscutum; Gtn, gastral tergite number; POL, the shortest distance between the posterior ocelli; OOL, the shortest distance between an eye and posterior ocellus. The type specimens were deposited in the Institute of Zoology, Chinese Academy of Sciences (IZCAS) and Nanjing Agricultural University (NAU).

### Molecular analysis

A total of 16 specimens of *Oomyzus* (ten *O. spiraculus* and six *O. scaposus*) were used for extractions of whole genomic DNA by using the DNeasy Blood & Tissue Kit (Qiagen) following manufacturer’s instructions. The primer pair LCO1490 (5′-GGTCA ACAAA TCATA AAGAT ATTGG-3′) and HCO2198 (5′-TAAAC TTCAG GGTGA CCAAA AAATCA-3′) (Folmer et al. 1994) were used to amplify the fragments of mitochondrial cytochrome c oxidase I (COI). All PCR procedures were performed using MyCycler Thermal Cycler (Bio-Rad, California, USA). The PCR reactions were carried out with Ex-Taq polymerase (Takara, Japan) under the following conditions: initial denaturation for 3 min at 94 °C, 35 cycles at 94 °C for 30 s, 52 °C for 40 s, and 72 °C at 30 s, followed by extension at 72 °C for 10 min. Sequencing was performed in both directions. Sequences of both directions were assembled and edited in Sequencher version 4.5 (Gene Codes Corporation, Ann Arbor, MI, USA) and aligned in BioEdit version 7.0.9.0 (Hall 1999). The COI matrix was translated into the amino acids in MEGA7.0 (Kumar et al. 2016) to check for stop codons. The Neighbor-Joining (NJ) phylogenetic tree based on the Kimura 2-parameter (K2P) distances was constructed by using MEGA7.0 with 1000 bootstrap replicates to generate support value for nodes.

Voucher specimens are deposited in the Nanjing Agricultural University. The obtained DNA sequences in this study have been deposited in Genbank (accession numbers MT259797–MT259812).

## Results

Two species of *Oomyzus* were reared from coccinellid pupae collected during field surveys in Jiangsu Province. One species was identified as *O. scaposus* and the other as a new species which is described and illustrated here.

A COI matrix containing 16 individuals of *Oomyzus* (ten *O. spiraculus* and six *O. scaposus*) with a length of 581 base pairs was generated after alignment and trimming, without insertion or deletion. Graphical representation of K2P distances between these 16 individuals based on COI is presented as an NJ tree in Figure 4. The minimum interspecific divergence (K2P distance) between *O. spiraculus* and *O. scaposus* is 7.6%. The maximum of intraspecific distance is 1.9% for *O. spiraculus* and 2.6% for *O. scaposus*.

### Systematics

#### Genus Oomyzus

Taxon classificationAnimaliaHymenopteraEulophidae

Rondani, 1870

F6ADF2C9-4F7A-526F-95C8-E21F76162F75

Oomyzus Rondani 1870: 141. Type species: Pteromalus gallerucae Fonscolombe, 1832, by monotypy.

##### Diagnosis.

Body black with metallic tinge varying from very weak to quite strong, never with pale markings. Malar sulcus straight or nearly so. Submarginal vein with 1 dorsal seta. MLM with 2–5 adnotaular setae; median line often absent, sometimes present, indistinct (e.g. *O. propodealis* Graham) or distinct (some species of *gallerucae*-group). Antenna with F1 often shorter than pedicel; male scape with a variable ventral plaque, from short to very long, and flagellum with rather short basal whorl of setae, or without whorls.

##### Remarks.

The genus *Oomyzus* is difficult to characterize and shares some features with some other genera of Tetrastichinae, such as *Baryscapus*, *Tetrastichus*, and *Quadrastichus*. *Oomyzus* usually has 3 or 4 adnotaular setae on MLM, but *Quadrastichus* has only 2; *Oomyzus* often has the female antenna with quadrate funiculars, but *Quadrastichus* has funiculars at least 2× as long as broad; *Oomyzus* has the female gaster shorter and less acute apically. Some *Oomyzus* species (e.g. *O. brevistigma*, *O. scaposus*, and *O. sokolowskii*) were originally regarded as *Tetrastichus* species. However, the characteristic Y-shaped carina formed by the paraspiracular carina and an additional carina running posterior-medially from the paraspiracular carina differentiates *Tetrastichus* from *Oomyzus*. The genus *Baryscapus* is distinguished from *Oomyzus* by the submarginal vein having 2 dorsal setae and a distinctly curved malar sulcus, although some species of *Oomyzus* occasionally have 2 dorsal setae on the submarginal vein (e.g. *O. sokolowskii*) and sometimes have a more or less curved malar sulcus (e.g. *O. pegomyae* Graham), which can be differentiated from *Baryscapus* by the combination of other diagnostic characters listed above. See also discussions in Graham (1991) and LaSalle (1994).

### Key to *Oomyzus* species similar to *O. spiraculus*

In this key both sexes are included and if ‘female’ or ‘male’ is not specified, then the features apply to both.

**Table:** 

1	Propodeum with distinct paraspiracular carinae (e.g. Fig. 3b)	**2**
–	Propodeum without paraspiracular carinae	**6**
2(1)	MLM without submedian line (e.g. Fig. 3b)	**3**
–	MLM with submedian line	**4**
3(2)	Fore wing with speculum large, extending some distance below marginal vein and sometimes reaching stigmal vein, usually more or less open below; propodeum medially 1.5–2× as long as dorsellum; antennal scape and pedicel testaceous	***O. sempronius* (Erdős)**
–	Fore wing with speculum small and hardly extending below marginal vein, closed below (Fig. 2h); propodeum medially relatively shorter, 1.1–1.2× as long as dorsellum (Fig. 3b); antennal scape and pedicel dark brown (Fig. 1b)	***O. spiraculus* Song, Fei & Cao**
4(2)	Fore wing with speculum relatively large, extending below marginal vein, open below (Yefremova and Yegorenkova 2010: figs 7, 9); face smooth	***O. hemerobii* Yefremova**
–	Fore wing with speculum small, not or only slightly extending to marginal vein, closed below; face weakly reticulate	**5**
5(4)	Propodeum relatively long, medially 3× as long as dorsellum; MLM with median line distinct (Yefremova and Yegorenkova 2010: fig. 16); male unknown	***O. rujumensis* Yefremova**
–	Propodeum short, medially about as long as dorsellum; MLM with median line indistinct	***O. propodealis* Graham**
6(1)	Anterior margin of clypeus with two distinct teeth or lobes; fore wing thickly or rather densely pilose, speculum very small; female antenna (Graham 1991: fig.192) short and stout, with pedicel distinctly longer than F1, F2 and F3 distinctly transverse; clava at most 2.6× as long as broad; male antenna having scape strongly swollen, at most 2× as long as broad, F2–F4 more than 1.5× as long as broad, each funicular with a compact subbasal whorl of long, dark setae (Graham 1991: fig. 210)	***O. incertus* (Ratzeburg)**
–	Anterior margin of clypeus with two minute tubercles; fore wing rather less thickly pilose, speculum slightly larger; female antenna (Graham 1991: fig. 195) with pedicel not or hardly longer than F1; clava 2.7–3.1× as long as broad; male antenna (Graham 1991: fig. 207) having scape normally swollen, about 2.5× as long as broad, F2–F4 subquadrate or slightly transverse, each funicular without compact subbasal whorls of long setae	***O. scaposus* (Thomson)**

### Species treatment

#### Oomyzus spiraculus

Taxon classificationAnimaliaHymenopteraEulophidae

Song, Fei & Cao sp. nov.

662DD96A-990C-5DAD-BA37-A36B180E1EFA

http://zoobank.org/B57F37F6-51E7-4EC3-A9DA-9541DBC2DC2E

Figures 1 , 2 , 3

##### Female.

**Figure 1.**
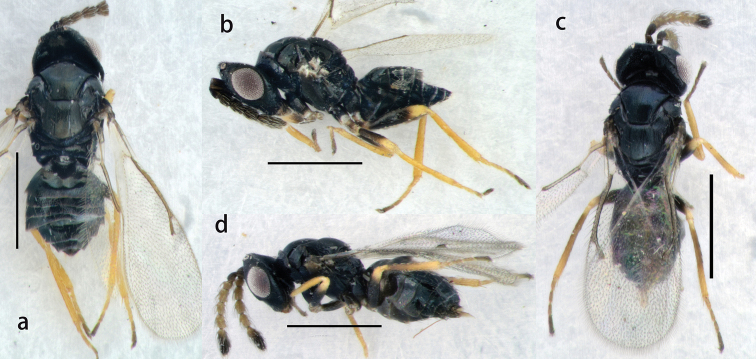
*Oomyzus spiraculus* Song, Fei & Cao. Female: **a** body in dorsal view **b** body in lateral view. Male: **c** body in dorsal view **d** body in lateral view. Scale bars: 0.5 mm.

**Figure 2.**
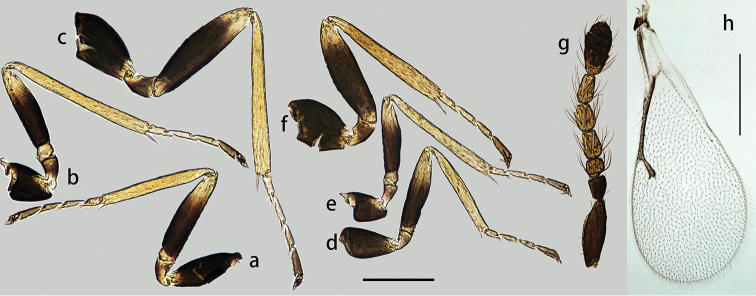
*Oomyzus spiraculus* Song, Fei & Cao. Female: **a** fore leg **b** mid leg **c** hind leg **h** fore wing Male: **d** fore leg **e** mid leg **f** hind leg **g** antenna. Scale bars: 0.2 mm.

Body length 1.2–1.4 mm. Body black with more or less dark green tinge (Fig. 1a, b). Antenna brown with apical scape and pedicel more or less light brown ventrally. Legs with coxae brown, tips of femora broadly brown and tibiae pale yellow; fore tarsus fuscous, turning to brown towards tarsomere 4; mid and hind tarsi with tarsomeres 1+2 pale yellow and tarsomeres 3+4 brown (Fig. 2a–c). Wings hyaline, with brown veins (Fig. 2h).

**Figure 3.**
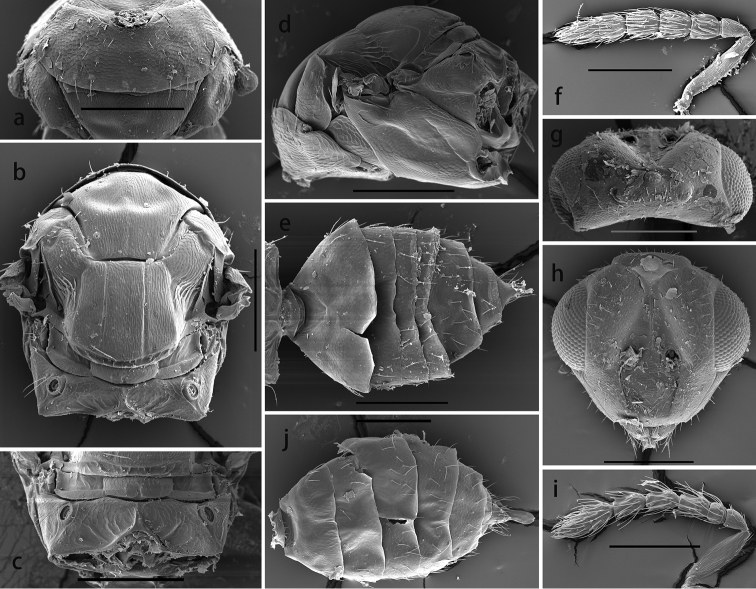
*Oomyzus spiraculus* Song, Fei & Cao. Female: **a** pronotum in dorso-anterior view **b** mesosoma in dorsal view **c** propodeum in dorsal view **d** mesosoma in lateral view **e** metasoma in dorsal view **f** antenna **g** head in dorsal view **h** head in frontal view. Male: **i** antenna **j** metasoma in dorsal view. Scale bars: 0.2 mm.

Antenna (Figs 1b, 3f) with 3 funiculars and 3 clavomeres; scape nearly reaching median ocellus, scape with raised and longitudinal reticulation; pedicel slightly shorter than F1, with raised striations; F1–F2 subequal in length and each about 1.4× as long as broad, F3 longer and more slender than F1 and F2, about 1.6× as long as broad; clava 0.7–0.8× as long as funicle, clavomeres decreasing in length, clavomere 3 with a short and indistinct terminal spine. Each flagellomere with longitudinal sensilla and apically with a circle of scattered, mushroom-shaped capitate peg sensilla; each flagellomere except clavomere 3 truncate apically.

Head slightly broader than mesoscutum, and very easily collapsing when dried. OOL 1.58× POL (0.70: 0.44) (Fig. 3g). Ocelli arranged in an obtuse triangle (Fig. 3g), almost in a line if head is collapsed. Ocellar triangle slightly raised. Head in anterior view 1.23× as broad as high (2.7: 2.2), with vertex convex (Fig. 3h). Frons with a short and narrow V-shaped frontofacial suture connecting to ocellar area; upper face with a thin and raised carina between depressed scrobes; head easily collapsed along frontofacial sutures and outer margins of scrobes (Fig. 3h). Face with longitudinal reticulation, scrobes with indistinct reticulation. Toruli inserted slightly above lower margin of eyes. Eyes with short and sparse white hairs, diameter larger than malar space. Malar space 0.8–0.9× as long as mouth opening, and malar sulcus more or less straight. Anterior margin of clypeus weakly bilobed (Fig. 3h).

Pronotum strongly sloping and almost invisible in dorsal view; pronotum distinctly reticulate, neck and collar not delimited, without posterior carina (Fig. 3a, b). Mesoscutum with engraved and longitudinal reticulation, notaular grooves deep and curved; MLM with 3 adnotaular setae, without median line or with a trace of median line only posteriorly, with posterior margin straight (Fig. 3b). Axillae strongly shifted forward, with engraved reticulation in anterior 2/3 and with strongly raised striations, like carinae, in posterior 1/3 (Fig. 3b). Scutellum convex in profile, slightly broader than long (1.3: 1.2), with engraved, longitudinal reticulation; scutellum with distinct submedian lines that are slightly nearer to sublateral lines than to each other, enclosed space between submedian lines 2.1–2.2× as long as broad; with two pairs of setae on scutellum, subequal in length, anterior pair situated slightly before middle and posterior pair situated near posterior margin; scutellum with depressed frenum, delimited by groove and scattered, irregular carinae (Fig. 3b, d). Dorsellum about 2.6× as broad as long, with coarser engraved reticulation than scutellum; slightly incised in middle of posterior margin; lateral panel of metanotum smooth, with a carina medially (Fig. 3b, c). Propodeum incised medially along anterior and posterior margins, thus shortest medially, medially slightly longer than dorsellum; propodeum with median carina, broadening caudad and then extending laterally; propodeum with paraspiracular carinae, median area, delimited by paraspiracular carinae and median carina, with slightly raised reticulation and with irregular oblique carinae posteriorly (Fig. 3b, c); spiracles with entire rim exposed; callus reticulate, with 4 setae. Lateral panel of pronotum, prepectus, mesepimeron and mesepisternum reticulate, except a small area between mesepimeron and mesepisternum that is smooth; acropleuron smooth; metapleuron reticulate (Fig. 3d).

Petiole short and hidden (Figs 1a, 3e). Gaster (Fig. 3e) 1.2–1.6× as long as broad and easily collapsed, especially Gt_2–4_ easily shrink or expand after death, and thus the relative length of mesosoma and metasoma is variable; gastral tergites each with weak raised reticulation; Gt_7_ with 4 cercal setae, the longest seta slightly longer than the other three setae that are subequal in length.

Legs (Fig. 2a–c) short and stout, with coxae, femora, and tibiae reticulate; tarsomere 1 of mid and hind legs almost as long as tarsomere 2. Fore wing (Fig. 2h) with postmarginal vein absent; submarginal vein with 1 seta on dorsal surface; speculum small, closed below, hardly extending below marginal vein.

##### Male.

Body length about 1 mm (Fig. 1c, d). Differs from female as follows. Antenna (Figs 2g, 3i) with 4 funiculars and 3 clavomeres, scape and pedicel black, funicle and clavomere 1 brownish yellow, remainder of clava black; scape with ventral plaque 0.57× total length of scape; F1 1.25× as broad as long, distinctly shorter than pedicel and F2; F2–F4 slender, subequal in length, 1.85–2× as long as broad; clava slightly broader than funicle, about 2.6× as long as broad, shorter than combined length of F3 and F4; each funicular with a compact subbasal whorl of dark setae which reach beyond tip of flagellomere attached to. Fore tarsi less infuscate (Fig. 2d). Gaster distinctly pointed apically (Fig. 3j).

##### Etymology.

From the Latin word *spiraculus* (spiracle), referring to the propodeum with paraspiracular carinae.

##### Type material.

***Holotype*** ♀, China, Jiangsu, Nanjing, Baima Agricultural Field of Nanjing Agricultural University, 30.V.2019, coll. Minghui Fei, *ex.* pupa of *Coccinella septempunctata* L. (IZCAS, IOZ(E)225734). ***Paratypes***: 5♀ 4♂, same data as holotype (IZCAS, IOZ(E)225735–IOZ(E)225742; NAU); 8♀ 3♂, China, Jiangsu, Nanjing, V.2019, coll. Haowu Hu, *ex.* pupa of *Coccinella septempunctata* L., lab reared on the pupae of *Coccinella septempunctata* (IZCAS, IOZ(E)225743–IOZ(E)225753; NAU).

##### Additional material.

3♀ 3♂ on slides, China, Jiangsu, Nanjing, V.2019, coll. Haowu Hu, *ex.* pupa of *Coccinella septempunctata* L., lab reared on the larva-pupa of *Coccinella septempunctata* (IZCAS).

##### Host and offspring information.

This species was reared as a gregarious endoparasitoid from pupae of *C. septempunctata*. In the laboratory, each instar of the host larva could be parasitized and would pupate successfully when provided with adequate number of aphids. The parasitoid offspring emerged from the host pupa, after a development time of 14–18 days. The brood size ranged from 4–23 after a single bout of parasitization, and the male number ranged from 0–3, mostly 2 (*n* =28). Another two common ladybird species, *Harmonia axyridis* Pallas and *Propylaea japonica* (Thunberg), were also included in the study and the result showed that the Japanese lady beetle, *P. japonica*, was a potential host.

##### Distribution.

China: Jiangsu.

##### Remarks.

*Oomyzus spiraculus* is one of five species of *Oomyzus* known to have propodeum with distinct paraspiracular carinae; the other four are *O. hemerobii*, *O. rujumensis*, *O. propodealis*, and *O. sempronius*. However, *O. spiraculus* is currently not supported to form a species group with any above-mentioned species by any other evidence. Morphological similarities and differences among these species are summarized in Table 1.

**Table 1.** Summary of morphological similarities and differences among *O. spiraculus* and some other *Oomyzus* species.

Species/Characters	*O. spiraculus*	*O. hemerobii*	*O. rujumensis*	*O. propodealis*	*O. sempronius*	*O. scaposus*
Paraspiracular carinae on propodeum	present	present	present	present	present	absent
Median line on MLM	absent	present	present	present	indicated in posterior half or absent	absent
Female tibiae	pale yellow	pale yellow	dark yellow	pale yellow	pale yellow	brown to black
Medially relative length of propodeum/dorsellum	at most 1.20×	1.5–2.0×	about 3.0×	about 1.0×	1.5–2.0×	at most 1.5×
Male F1	transverse, shorter than pedicellus	subquadrate, shorter than pedicellus	unknown	longer than broad, as long as pedicellus	longer than broad, shorter than pedicellus	subquadrate, shorter than pedicellus
Cercal setae	one slightly longer than the other three	unavailable	unavailable	longest one nearly 2× length of next longest, kinked	unavailable	subequal
Face	sculptured	smooth	sculptured	sculptured	sculptured	sculptured
Speculum of fore wing	closed below, small, not extending below marginal vein	closed below, relatively large, extending along marginal vein	closed below, small, not extending below marginal vein	closed below, small, not extending below marginal vein	open below, large, extending some distance below marginal vein	closed below, small, not extending below marginal vein
Color of antennal scape in female	mainly brown, paler apically	yellow dorsally, brown dorsally	yellow	black	testaceous	fuscous to brown
Color of antennal pedicel in female	mainly brown, paler ventrally	yellow dorsally, brown dorsally	dark brown	black	testaceous	fuscous to brown

In addition, the relatively short propodeum with paraspiracular carinae and the pale-yellow tibiae differentiate *O. spiraculus* from *O. scaposus* reared from the same host (Table 1). Male tibiae of *O. spiraculus* are mostly pale yellow as the female, sometimes slightly infuscate dorsally.

**Figure 4.**
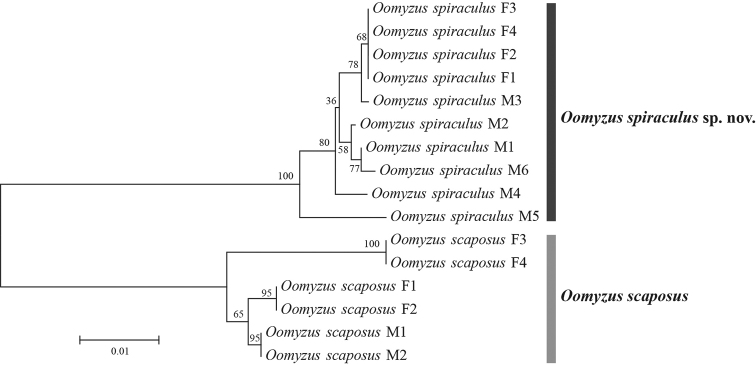
NJ tree of COI sequences from 16 *Oomyzus* specimens using K2P distances. Numbers above/below nodes represent bootstrap values.

## Discussion

*Coccinella septempunctata* is a dominant predator attacking aphids in agroecological ecosystems and has great potential as a biological control agent in the development of a green agricultural economy. Prior to this study, quite a few Chalcidoidea parasitoids of *C. septempunctata* have been reported, including species belonging to Encyrtidae, Eulophidae, and Pteromalidae. *Homalotylus* spp. (Hymenoptera: Encyrtidae) seem to be especially well associated with *C. septempunctata* (Noyes 2019). In China, *Oomyzus scaposus* and *Homalotylus flaminius* (Dalman) are the most common parasitoids of *C. septempunctata* (Jing and Huang 2002; Song et al. 2017). This study demonstrates that *O. spiraculus* is a new parasitoid species of *C. septempunctata*, and it provides an ideal model system together with *O. scaposus* for further studies of interactions with *C. septempunctata*, as well as competition among themselves. In addition, the description of this new species will facilitate the discussions of phylogenetic relationships between close species of *Oomyzus* and the divergence and speciation of parasitoids in the same niche.

## Supplementary Material

XML Treatment for Oomyzus

XML Treatment for Oomyzus spiraculus
